# Comparison of dried and liquid direct-fed microbial (MYLO) on liveweight gain and carcass performance in feedlot cattle

**DOI:** 10.1093/tas/txag033

**Published:** 2026-03-22

**Authors:** Jane C Quinn, Paul M V Cusack

**Affiliations:** School of Agricultural, Environmental and Veterinary Sciences, Charles Sturt University, Wagga Wagga, NSW 2678, Australia; Gulbali Institute for Agriculture, Water and the Environment, Charles Sturt University, Wagga Wagga, NSW 2678 Australia; School of Agricultural, Environmental and Veterinary Sciences, Charles Sturt University, Wagga Wagga, NSW 2678, Australia; Australian Livestock Production Services, Cowra, NSW 2794, Australia

**Keywords:** cattle, carcass, direct-fed microbial feedlot, *Lactobacillus*, liveweight

## Abstract

Direct-fed microbials (DFMs) are increasingly being used as supplements to support productivity and gut health in ruminants. One supplement available commercially is MYLO^®^ (Terragen, Coolum Beach, Australia), a liquid DFM supplement (‘Liquid’) (Terragen, Coolum Beach, Australia), that contains three bioactive microbial strains: *Lacticaseibacillus paracasei, Lentilactobacillus buchneri*, and *Lacticaseibacillus casei*. To determine equivalence of the commercially-available Liquid MYLO DFM supplement (‘Liquid’) to a new dried MYLO formulation, a study was undertaken using purebred Angus steers (*n* = 264) fed a high-grain diet supplemented with 1 × 10^10^ Colony Forming Units (**CFU**/hd/day, X1 MYLO), 2 × 10^10^ CFU/hd/day (X2 MYLO), or liquid MYLO (Liquid) compared to a control diet containing no DFM (Control). Steers were fed a total mixed ration (**TMR**) with increasing grain inclusion (starter ration, 58% grain; intermediate, 68% grain) with all steers on finisher ration (78% grain) by day 21. Steers were grain fed for 106 days prior to slaughter at a commercial processing plant. Steers were allocated to pens (22 head/pen, 3 pens/treatment) using a randomised block design, and production, health, and carcass data were analysed. Data were analysed using generalised mixed linear models with pen as the experimental unit. Liveweight gain, feed efficiency, health and carcass parameters were compared between MYLO groups and an untreated control cohort. Results showed that steers supplemented with X2 MYLO dried formulation showed a trend towards an increased liveweight with significance at 82 and 106 DOF (*P* = 0.014 and 0.032, respectively). No difference was observed in overall Average Daily Gain (**ADG**) (*P* = 0.997) or Gain to Feed Ratio (**G : F**) (*P* = 0.760) between treatments despite X2 MYLO trended towards greatest G : F ratios (Control G : F, 0.160; X2 MYLO, 0.163; liquid MYLO, 0.165). Hot standard carcass weight (**HSCW**) trended higher in X2 MYLO steers, resulting in an increased carcass value of $41 Australian Dollars (**AUD**)/head. *Longissimus dorsi* eye muscle area, as measured between the 12-13^th^ rib, was increased in X2 and Liquid MYLO DFM groups compared to controls (*P* = 0.037 and 0.008 respectively). Overall, the *Lactobacillaceae*-based X2 MYLO dried supplement showed increases in liveweight gain, carcass weight, and muscle area compared to low-dose dried or liquid formulations.

## Teaser

A dried, concentrated direct-fed microbial supplement increased liveweight gain and improved carcass performance in feedlot steers.

## Graphical Abstract

**Graphical Abstract:**
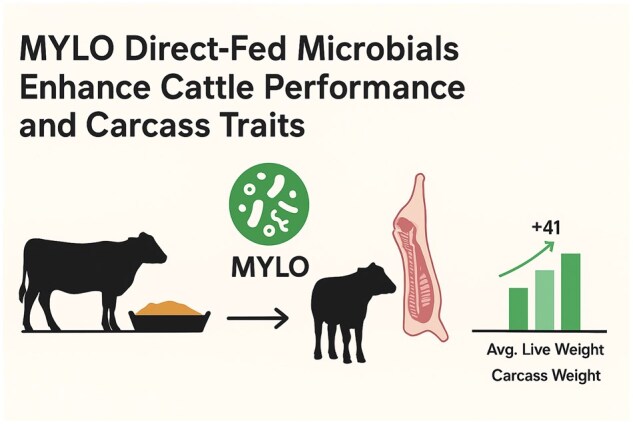

## Introduction

Direct-fed microbials (**DFM**) are supplements that contain live microorganisms that are thought to confer positive health benefits in humans and animals. DFMs have been investigated for several decades due to their potential to enhance growth, health, and productivity in cattle in both beef and dairy systems (Morelli and Capurso 2012). There have been suggestions that DFMs could also confer improved public safety through suppression of zoonotic bacteria in cattle (Stephens et al. 2007; Cull et al. 2015; Vipham et al. 2015), although strong evidence to support their use in replacing antimicrobials is still lacking (Cusack 2024). In ruminants, particularly calves and lot-fed beef cattle, using DFMs may support digestive health and mitigate common metabolic and infectious challenges associated with intensive feeding practices (Krehbiel et al. 2003). Young ruminants have an immature digestive system and a developing microbiota, making them susceptible to gastrointestinal issues, which DFMs may help alleviate by establishing a stable and beneficial gut environment (Signorini et al. 2012). Certain DFM strains, such as *Lactobacillus*, *Bifidobacterium*, and *Saccharomyces cerevisiae*, are effective in modulating gut microbiota, improving nutrient absorption, and enhancing immune function (Uyeno et al. 2015). For instance, *Lactobacillaceae* spp. have been shown to reduce the incidence of diarrhea in calves, a prevalent issue that hinders early growth and increases mortality risks (Malmuthuge and Guan 2017). In lot-fed beef cattle, high-grain diets necessary for rapid growth can lead to digestive disorders like acute and subacute acidosis and bloat; it has been suggested DFMs may help balance rumen pH and reduce these risks (Beauchemin et al. 2003).

DFMs are suggested to maintain a stable microbial environment in the rumen, thus optimising digestion, enhancing feed efficiency, and increasing growth performance in grain-fed cattle (Ghorbani et al. 2002, Silva et al. 2024). The mode of action varies by DFM species; for example, *Saccharomyces cerevisiae* is proposed to stabilize ruminal pH by consuming lactic acid, making it particularly valuable for cattle on high-concentrate diets (Chaucheyras-Durand et al. 2008), with the potential to mitigate subacute ruminal acidosis, a common issue in lot-feeding systems. Interactions between the rumen microbiome profile and production outcomes have also been identified in feedlot steers, with relationships to both liveweight gain and marbling characteristics correlated with some bacterial and protozoal families (Sato et al. 2024).

Supplements designed to interact with, or attenuate rumen microbial populations, and their bioactivity, are an increasing focus of the animal health industry with an ever-expanding number of rumen-modulating products available on the market (McCann et al. 2017). How the rumen microbiome responds to use of pre- and DFMs, and how host individuality in microbiome dynamics, species redundancy and/or resilience contributes to stability or modification of microflora subpopulations is still largely unknown, although increasing numbers of studies are investigating environmental/host/microbiome interactions (Xue et al. 2020; Maslen et al. 2022) mining the rumen microbiome for genera of key importance, such as the H_2_-oxidizing, CO_2_-reducing acetogens and methanogens (McCann et al. 2017) as well as examining rumen microbiome changes in response to lactobacilli-based DFM supplementation (Campbell et al. 2025).

Despite these promising benefits, the variability in probiotic strains and dosing strategies has led to inconsistent results across studies (Jami et al. 2013; Cusack 2024). Direct-Fed microbial efficacy can be influenced by factors such as breed, diet, environmental conditions, dietary composition, and the unique microbial ecosystem as well as their persistence in the rumen (Dill-McFarland et al. 2019; Monteiro et al. 2022; Susanto et al. 2023; Bu et al. 2025), with early programming identified to play a key role (Furman et al. 2025). Various protocols have been used for DFM administration in cattle that have been tailored to specific stages of development and diet types for this reason (Velasquez-Munoz et al. 2022; Treon et al. 2024) leaving a confusing number of options for producers.

Despite a significant body of research, there is still limited evidence of efficacy for DFMs as a promotor of growth in feedlot steers. To address this, the current study investigated the use of a commercially available DFM (MYLO^®^ liquid supplement, Terragen Pty Ltd, Coolum Beach, Australia) containing a mixed population of *Lacticaseibacillus paracasei*, *Lentilactobacillus buchneri* and *Lacticaseibacillus casei* using two formats (dried and liquid) at two dose rates (X1 Liquid, 1X dried and 2X dried) in cattle fed a high grain ration in a feedlot for 106 days. Supplement was added to the feed daily and performance outcomes were compared to cattle receiving no DFM supplementation.

## Materials and methods

### Animals and experimental design

All procedures and protocols in this study complied with the Australian Code of the Care and Use of Animals for Scientific Purposes (2013) and was approved by the Charles Sturt University Animal Ethics Committee (Protocol A24192). The cattle owner provided informed consent prior to the trial’s initiation. The experiment used a randomised, controlled, replicated, block design, with treatment as the block and pen as the experimental unit.

Pure-bred Angus steers were sourced from four vendors in central New South Wales, Australia (*n* = 277), and arrived at the experimental site between 29th July and 2nd August 2024. All steers were retained in their arrival cohorts and placed directly onto cereal hay and ad-libitum water. Arrival weights were taken after a period of settlement (30 minutes). After weighing, steers were moved to temporary holding pens in their arrival cohorts and placed on ad-lib lucerne hay, cereal hay and water until induction. Arrival weight ranged from 365–483 kg liveweight (**LW**) with a mean of 414 kg. Steers were stratified to pen by arrival weight and allocated to pens (12 pens total; 3 pens per treatment). All cattle were weighed again at induction (Day 0) and total and mean pen liveweights reviewed to ensure intake weights were balanced between pens and across treatments. Induction LW by pen was 424.92 kg, with a range of 424.54–424.96 kgLW (ANOVA: *F = *0.583. *P* = 0.642). Total pen weight was also checked for balance between pens and treatments (Mean total pen weight by treatment: 9348 kgLW, range 9318–9349 kgLW (ANOVA: *F = *0.583. *P* = 0.642). Average Daily Gains (**ADG)** were assessed at each weigh point, with and Gain to Feed ratios (**G : F**) calculated relative to total DMI.

### Cattle processing and management

Cattle were inducted into the trial on 5th August 2024. During the presentation at the crush, each steer was identified by a unique visual ID tag, and the EID was recorded. All cattle received the following treatments: TasVax 8 in 1 clostridial vaccine for cattle (Coopers Animal Health), Rhinogard Bovine Herpesvirus 1 Live Intranasal Vaccine for Cattle (Zoetis, Parsippany, NJ, USA, 2 mL, intranasal) and Bovishield MH One *Mannheimia haemolytica* Vaccine for Cattle (Zoetis, 2 mL, subcutaneous), treated with Cydectin Pour-on for Cattle (Virbac Australia, 5 mg/L moxidectin, 50 mL) and drenched with an oral flukicide Flukare C (Virbac Australia, Milperra, New South Wales, Australia, 120 g/L triclabendazole, 50 mL).

Cattle remained in their assigned pen for the duration of the study unless removed for health conditions requiring intervention. All cattle in each pen were fed the same diet under the same environmental conditions and weather events. Cattle were fed twice daily using a clean bunk feeding program where trained feedlot staff recorded orts daily to determine ration increases relative to pen intakes with a target of crumbs present at bunk calling. Diets were fed as a 60:40 allocation, a.m. to p.m. feed.

Curfewed LW’s were obtained prior to morning feeding on d0 (day of induction), d14, d28, d42, d56, d82 and d106 using a Clipex HD2000 pneumatic crush with autodrafter and EID reader and using a Tru-Test XR5000 data reader for data capture and collection. Cattle were weighed in pen cohorts in order of feed delivery, specifically: Control, then MYLO x1, MYLO x2, and finally MYLO Liquid pens. Any animals removed from the trial, and all treatments given during the trial, were recorded.

### Rations

Diet compositions are shown in Table 1. Feed testing was performed weekly for the duration of the trial to ensure consistency. In addition to the base diet, limestone and a premix containing vitamins (A, B1, D, E), trace minerals (cobalt, copper, iron, selenium, zinc, manganese, iodine) and monensin (Rumensin, Elanco Australia Pty Ltd, Australia; 25 g/T) were added to each feed at mixing. Starter ration was delivered for 11 days, followed by an intermediate ration for 10 days, with steers placed on a finisher diet on 26th August 2024. As-fed amounts were calculated daily on a rising plane of intake, based on bunk calls and assumed weight gain, rising from 8.82 kg/h/d at D0 to a maximum of 18.42 kg/h/d at Day 106. The Total Mixed Ration (**TMR**) met or exceeded the National Feedlot Accreditation Scheme (**NFAS)** grain composition and metabolizable energy (ME) requirements for feedlot cattle. Bunk calling occurred between 6.30 and 7.30 a.m. daily, with any refusals collected and weighed for calculation of total dry matter intake (DMI) per pen. Total as-fed total rations (kg) were calculated based on daily total pen allocations minus refusals. Water was available *ad-libitum* in all pens with troughs cleaned every 3 days, or more frequently if required. A composite ample of the control ration was collected weekly for feed quality analysis to ensure comparability of composition across the duration of the trial.

**Table 1:** Diet composition and nutritional profile of starter, intermediate and finisher diets.

Ingredient, % of TMR^2^	Diet^1^		
	Starter	Intermediate	Finisher
**Maize**	36	36	36
**Lucerne Hay**	15	10	–
**Oaten hay**	15	10	10
**Canola meal**	12	12	12
**Barley**	10	20	30
**Premix (general)**	9	9	8
**Vitamin and mineral premix^3^**	2	2	2
**Limestone**	1	1	1
**Rumensin**	0.027	0.027	0.027
**Analysed composition, % DM^3^**			
**Neutral Detergent Fibre**	27.1	24.2	22
**Starch**	27.59	31.88	37.94
**Crude protein**	13.3	13.6	14.3
**Total dry matter**	89.6	89.3	91.0
**Metabolizable energy (MJ/kg)**	10.7	12.4	12.9

1 Steers were fed the same diet except for inclusion of experimental treatments.

2 Traditional mixed ration.

3 Premix containing cobalt, copper, iron, selenium, zinc, calcium, phosphorus, magnesium, potassium, sodium, manganese, Vitamin A, B1, D, and E.

4 Based on composited TMR sample analysed by New South Wales Department of Primary Industries Feed Laboratory, Wagga Wagga, Australia.

All cattle were acclimatised overnight before initiating control or treatment diets in a starter ration on 6th August. All pens were fed a morning (8 a.m.) and afternoon (3 p.m.) feed with a 60:40 split. Control pens were fed TMR without the addition of any DFM supplement. Treatment pens were fed TMR plus the required DFM supplement with the full dose delivered in the morning feed only. All MYLO™ supplements contained *Lacticaseibacillus paracasei*, *Lentilactobacillus buchneri* and *Lacticaseibacillus casei* in 1 × 10^10^ CFU concentrations, were prepacked into sealed foil sachets containing a single daily dose and stored at 4°C prior to use. The dried supplement contained the bacteria plus a carrier (maltodextrin), while the liquid supplement contained the bacteria in 5% molasses in dechlorinated food-grade water.

MYLO supplements (dried and liquid) were prepared daily and incorporated into the morning (8 a.m.) ration. MYLO dried supplements were reconstituted with 6600 mL of lukewarm dechlorinated water immediately prior to inclusion in the ration and fed at rates of 1 × 10^10^ CFU/hd/d for (300 mL/hd/d, X1 MYLO) or 2 × 10^10^ CFU/hd/d (300 mL/hd/d, X2 MYLO). MYLO liquid supplement was provided at a volume of 10 mL/hd/day containing 1 × 10^10^ CFU/hd/d daily diluted into a total volume of 6600 mL lukewarm dechlorinated water (300 mL/hd/d, Liquid MYLO). Rations were prepared in bulk per treatment, with the total ration per treatment and per pen calculated daily based on bunk calls. Inclusion rates of supplement were adjusted against any animal removals to ensure consistent dosing.

### Health records and treatments

Cattle health was managed according to standard feedlot treatment protocols. Pens were checked twice daily for identification of sick or injured stock, and steers showing clinical signs sufficient to warrant treatment were removed for inspection and/or management. All records of treatments for respiratory disease, lameness, gastrointestinal disorders, injury and bulling were collected for the duration of the trial.

### Treatment withdrawals

Cattle were withdrawn from the study and removed to a separate feeding pen 1) if they received 3 or more antibiotic treatments for an identified medical condition, or 2) if they were moved to the hospital pen for more than 2 days. In total nine steers were excluded from the trial during the feeding period (Pen 3, X2, *n* = 1; Pen 7, X1 *n* = 1, Pen 8, Liquid, *n* = 1; Pen 9, Control, *n* = 3, Pen 10, X2, *n* = 1; Pen 11, Control *n* = 1) with one found dead by misadventure (Pen 3, X2, *n* = 1) and one animal removed as a buller at day 96 on feed (Pen 12, Liquid, *n* = 1). This steer was maintained on the treatment diet until the end of the feeding period and was therefore included in the final analysis. The number of cattle remaining on feed per treatment at the end of the feeding period (106 days on feed) was 255: Control; *n* = 62, MYLO X1; *n* = 65, MYLO X2; *n* = 63, MYLO Liquid; *n* = 65.

### Processing and carcass measurements

Cattle underwent a final weighing at exit and were transported to a local processing facility (10 km distance) and processed on the day following transport. All cattle were slaughtered using humane practices and in accordance with Australian government requirements for meat processing for human consumption. An observer was stationed at the body number station to cross-check electronic ear tag, visual ID and body number. After slaughter carcasses were assessed for antemortem disease or defects by a trained meat inspector and defects or condemnations were recorded.

After chilling, carcasses were assessed by trained meat inspectors to determine P8 fat depth and marble score. Grading data was correlated from both sides of the carcass to yield a final Meat Standards Australia score. Carcass data included dentition, fat depth, fat colour, meat colour, Meat Standards Australia (**MSA**) marble score, ossification score, P8 fat depth, *longissimus dorsi* eye muscle area (cm^2^), measured between the 12-13th rib, hot standard carcass weight (**HSCW**), lean muscle yield, and carcass value in Australian dollars (AUD). All steers were classified as Hormone Growth Promotant (**HGP**)-free Angus steers at grading.

### Statistical analysis

Data were visually assessed for normality using Q : Q and P: P data charts and non-parametric tests applied where data were not normally distributed. Data were analysed using a randomised block design with the pen as the experimental unit and treatment as the block. Time was considered a fixed effect, with pen and origin as random effects. Where cattle were removed from the study, pen means and DMI were adjusted accordingly to give a ‘rejects out’ analysis. Liveweight, ADG and G : F ratio were calculated and compared between treatments and across time. Assumptions of difference were tested using ANOVA in the first instance, with a general mixed model for repeated measures used to analyse LW gain, ADG and G : F over time with treatment as a fixed effect, with pen and origin as a random effects. Within and between treatment effects were compared and where Mauchly’s test of sphericity was not met, Greenhouse-Geisser correction was applied. Tests of within-subject comparisons included time and treatment*time. Final exit ADG, LW gain and G : F were compared using multi-way ANOVA on a rejects out basis and with pairwise comparisons undertaken, using Bonferroni adjustment. Health data, including the incidence of lameness and respiratory disease, were analysed using ANOVA and for disease*treatment interactions. Carcass data were analysed using multivariate mixed linear modelling with diet treatment as a fixed effect and pen and origin as covariates. Pairwise comparisons of estimated marginal means were also performed to assess treatment differences. Where normality of distribution was not met, non-parametric analysis used Pearson’s Rank Sum or Kruskal-Wallis tests. Significance in all cases was considered at *P* < 0.05 with statistical trends considered at 0.05 < *P* ≤ 0.1. All data was analysed using IBM SPSS Statistics for Windows (version 29.0, IBM Corporation, Armonk, NY, USA).

## Results

### Growth performance

Of the 264 head inducted into the trial, 255 remained on feed at the end of the feeding period, with 8 steers removed under exclusion criteria for antibiotic treatment, and one death by misadventure. Liveweights were balanced at entry to the trial to ensure comparative entry weights between the treatment groups. Growth performance and carcass characteristics, including mean total carcass values, for all steers that completed the feeding period, are shown in Table 2.

**Table 2:** Mean performance traits of feedlot steers by treatment for control, MYLO liquid or dried supplement treatment groups. Data is shown as mean ± SEM.

	Control^1^	X1 MYLO^1^	X2 MYLO^1^	Liquid MYLO^1^	TRT *P*-value
**Initial LW, kg^2^**	424.92 ± 1.60	424.97 ± 1.07	426.45 ± 1.60	423.55 ± 1.49	>0.05
**Exit LW, kg**	659.29 ± 3.26	655.49 ± 4.22	668.11 ± 4.26	654.31 ± 3.44	>0.05
**Overall ADG, kg/h/d**	2.18 ± 0.01	2.17 ± 0.05	2.28 ± 0.04	2.17 ± 0.03	>0.05
**G:F ratio, ADG/DMI^3^**	0.160 ± 0.006	0.161 ± 0.003	0.163 ± 0.004	0.166 ± 0.003	>0.05
**DMI, % LW**	0.202 ± 0.02^a,b,c^	0.205 ± 0.02^a,b,c^	0.208 ± 0.03^a,b,c^	0.200 ± 0.02^a,b,c^	>0.05^a,b,c^
					**0.048^a,b,c^** ^**,^b^**^
**DMI, kg/h/d**	13.6 ± 0.44	13.5 ± 0.53	13.9 ± 0.11	13.1 ± 0.03	>0.05

1 Control *n* = 62; X1 MYLO *n* = 65; x2 MYLO *n* = 63; Liquid MYLO *n* = 65.

2 All groups *n* = 66.

3 Gain to feed ratio.

4 Dry matter intake.

a,b,c Within row means with different superscripts differ (*P* < 0.05).

Significant *P* values are shown in bold.

**Figure 1:**
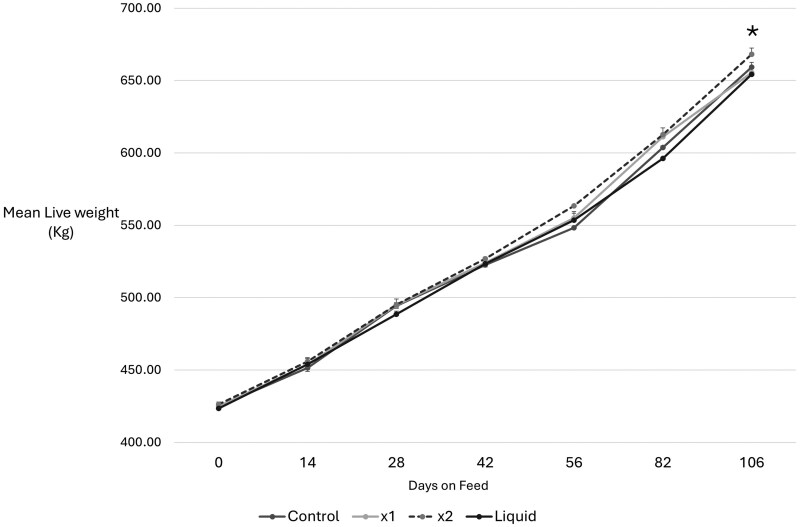
Mean live weight (kg) by treatment at day of induction (day 0) and after 14, 28, 42, 56, 82, and 106 d on feed for feedlot steers fed a traditional mixed ration with or without MYLO DFM supplements. Treatments: Control, dried X1 or X2 MYLO and liquid MYLO formulation. *N* = 3 in all cases. Data is shown as mean ± SEM. Significant differences between treatments are noted. *P = <0.05.

When total liveweight gain was considered (D106), univariate analysis showed that LW did not differ between treatments at exit (*F = *1.581, *P* < 0.195) although pairwise comparison indicated MYLO X2 showed a positive trend (*t = *1.948, *P* < 0.052), (Fig. 1, Tables 2 and 3). No difference was observed in exit liveweight performance between controls and X1 or Liquid treatment groups.

**Table 3:** Mean percentage disease prevalence in feedlot steers fed daily with a total mixed ration with or without direct fed microbial treatments.

Disease, % (*n*)	Control^1^	x1 MYLO^1^	x2 MYLO^1^	Liquid MYLO^1^	TRT *P*-value
**Lameness, %**	12.2 (8)	10.6 (7)	13.6 (9)	6.0 (4)	>0.05
**Respiratory disease, %**	34.8 (23)^a^	50.0 (33)^b^	37.9 (25)^a^	27.3 (18)^a,b,c^	0.899^a^
					**0.046** ^a,b^
					**0.025** ^a,b,c^
**Injury, %**	1.5 (1)	–	1.5 (1)	1.5 (1)	>0.05
**Gastrointestinal disorder, %^2^**	1.5 (1)	–	3.0 (2)	–	>0.05
**Buller syndrome, %**	–	–	–	1.5 (1)	N/A

1 All groups *n* = 66.

2 Includes acidosis.

a,b,c Within row means with different superscripts differ (*P* < 0.05).

Significant *P* values are shown in bold.

To investigate interactions between time on feed and treatment, a repeated measures ANOVA was undertaken with time and treatment as fixed effects (Table 2, Table S1). Mauchly’s test of sphericity was not met (*P <* 0.001) so a Greenhouse-Geisser correction was used (*P* = 0.478). Post hoc analysis using Bonferroni adjustment showed that time * treatment exerted a significant effect (*F = *133.779, *P* < 0.001). A test of within subject contrasts indicated significant at both quadratic 5th order levels (*F = *13.761, *P* < 0.001) indicative of the significant variation in LW gain observed over the time on feed suggesting interactions between time and diet for LW gain. Levene’s test of equality showed no significant impact of time on variance by DOF (D0: *F = *0.703; *P* = 0.511; D14: *F = *1.088; *P* = 0.335; D28: *F = *0.538; *P* = 0.657; D42: *F = *0.1.259; *P* = 0.289; D56: *F = *1.305; *P* = 0.273; D82: *F = *0.165; *P* = 0.920; D106: *F = *0.866; *P* = 0.459) supporting a between subjects analysis. Pairwise analysis showed a significant difference between mean exit liveweight of the Liquid and X2 MYLO treatments (*P* = 0.042, Fig. 1, Table S1) with no significant difference between the other treatment groups.

Analysis of between subject interactions between time and treatment identified key interactions at D82 and D106 for X1 MYLO and X2 MYLO treatment groups (D82: X1 MYLO *t = *0.2019; *P* = 0.045; X2 MYLO *t = *2.845.; *P* = 0.014; D106: X2 MYLO *t = *2.152.; *P* = 0.032) indicative of the marginal increase in exit LW observed in the X2 MYLO group compared to other treatments (Fig. 1). Neither pen nor origin exerted an effect on liveweight gain over time (Time*Pen*treatment, *F = *0.663; *P* = 0.416), nor did origin affect treatment outcomes (Time*origin*treatment, *F = *0.616; *P* = 0.433).

**Figure 2:**
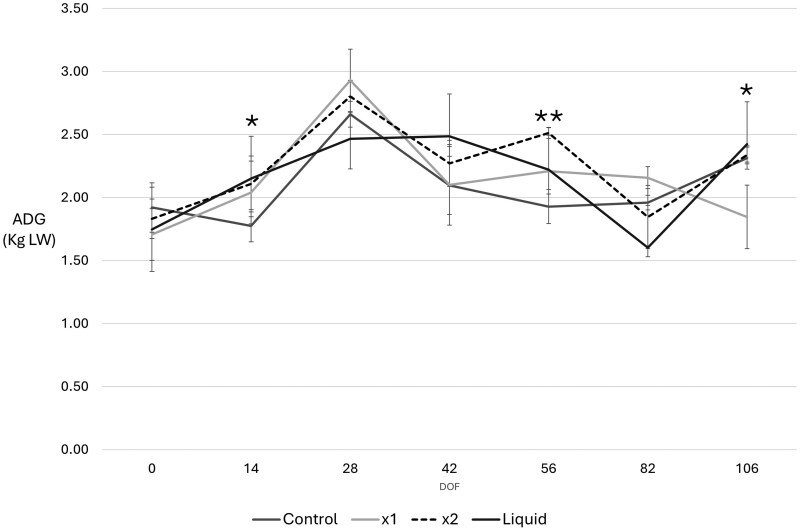
Mean average daily gain (kg/h/day) by treatment at day of induction (day 0) and after 14, 28, 42 and 56, 82 and 106 days on feed for feedlot steers fed a traditional mixed ration with or without MYLO DFM supplements. Treatments: Control, dried MYLO at X1 and X2 concentrations or liquid MYLO formulation. *N* = 3 in all cases. Data is shown as mean ± SEM. Grand mean is shown as a black line and significant differences between treatments are noted. **P < 0.05; ***P < 0.001.

A similar analysis was conducted for Average Daily Gain (ADG). Univariate analysis showed no difference in ADG between treatments (*F = *1.092, *P* = 0.353, Mean 2.189, CI 2.147–2.355) with no skewness or inequality of variance in the data (Levene’s test: *F = *1.28, *P* = 0.257). To consider impacts of time* treatment on ADG, a repeated measures analysis was undertaken using Greenhouse-Geisser and Bonferroni adjustment, which showed a significant variance in ADG between groups over time (*F = *21.599, *P* < 0.001). Pairwise comparison showed no differences between treatment groups for any combination (*P* = 0.703–1.0). When individual timepoints were considered, highest ADGs were observed in X1 and X2 MYLO groups after 28 days on feed (X1 MYLO ADG 2.92 kg/head/d SD1.5; X2 MYLO ADG 2.78 kg/head/d SD 0.98), although these were not significantly different to controls (*P* = 0.082) (Fig. 2, Table S2).

Within subjects contrasts indicated variance in the data over time (Fig. 2, F = 2.193, *P* = 0.035) with time*diet exerting an effect (*F = *3.039, *P* < 0.002). Between subjects analysis also reached significance (*F = *2.645, *P* = 0.50) although this significance was lost overall when pairwise comparisons were considered (Table S2).

**Figure 3:**
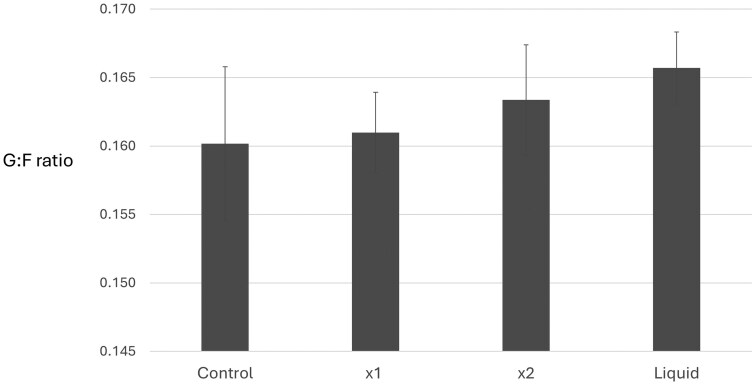
Gain to feed ratio (G:F) by treatment for feedlot steers fed a traditional mixed ration with or without MYLO DFM supplements after 106 days on feed. Treatments: Control, dried MYLO at X1 and X2 concentrations or MYLO liquid formulation. Data is shown as mean ± SEM. Control, *n* = 62; X1, *n* = 65; X2, *n* = 63 and liquid, *n* = 65.

Gain: Feed ratio was considered using univariate and repeated measures analysis. Mean G : F did not show a significant difference between treatments (*F = *0.0.393, *P* = 0.760) and met Levene’s test of equality: *P* = 0.655). Liquid and MYLO x2 supplemented groups showed a numerically lower G : F over the duration of the trial, but this trend was not significantly different to controls (Fig. 3, Table 2). A marginal increase in DMI % LW was also observed in the X2 MYLO group compared to controls (*P* = 0.048, Table 2), consistent with a marginally greater DMI in this group over the feeding period.

### Prevalence of disease conditions

**Figure 4:**
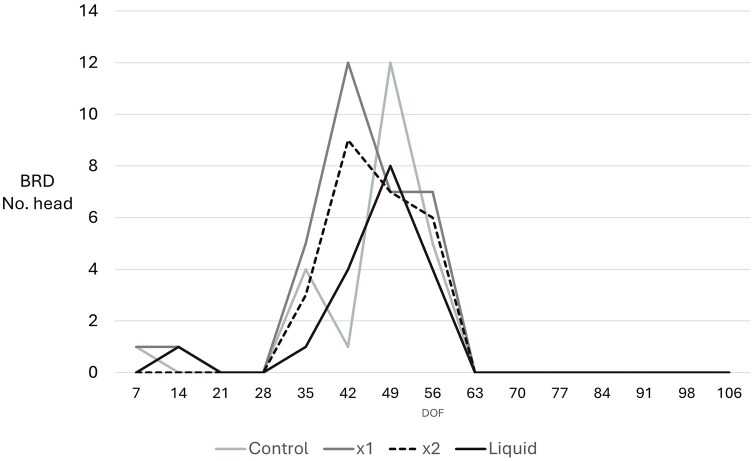
Total incidence of respiratory disease in cattle fed a DFM-containing diet compared to controls over a 106-day feeding period. Exclusions are included, *n* = 66 in all cases.

Daily records of pull reasons were examined for all treatment groups and were recorded daily, specifically lameness, respiratory disease, gastrointestinal disease, injury and buller syndrome (Table 3). Where the incidence of disease was sufficient in number across time and between treatments for statistical analysis (lameness and respiratory disease, Table 3), no significant difference was observed between the numbers of steers presenting for treatment between MYLO treatment groups or controls or across time. When total number of cases were compared using a deads and rejects in comparison between DOF, Pearson’s correlation identified a trend towards a significant difference between incidence of BRD occurring between D42 and D49 on feed (mean total cases per treatment D42: 6.5 ± 4.9; D49: 8.5 ± 4.9, *P* = 0.051) showing the peak of respiratory disease incidence. Most cases presented between D28 and D56 on feed (Fig. 4), with no further cases pulled for treatment after D63. The 1X MYLO group showed a significant increase in respiratory disease cases compared to all other MYLO groups (*P* = 0.046, Fig. 4), with the Liquid MYLO group showing significantly fewer respiratory disease pulls (*P* = 0.025, Table 3). When disease incidence was considered as a covariate factor for overall liveweight gain, no significant effect of disease was identified for exit LW (Exit LW: *P* = 0.914; ADG: *P* = 0.910).

### Carcass performance

Univariate analysis showed no difference between treatments for HSWC, lean meat yield, ossificiation, carcase pH, rib fat depth, MSA marbling score or overall MSA score (Table 4). All bar six carcasses graded with an MSA score of 60.00 or above, with the highest score being 67.32 (a control treatment steer). Only two outliers were noted with scores below the 3^rd^ quartile, one in the control group and another in the MYLO liquid group. Although not reaching significance, HSCW of carcasses from the X2 MYLO treatment group showed the heaviest carcase weights (*P* = 0.077, Table 3) consistent with their greater LW at exit (Table 2). Coincident with an overall increase in HSCW, mean carcass value was numerically greatest in the X2 MYLO treatment group (Table 3, Table S3). MSA marble score was observed to trend higher in the X1 MYLO group compared to other cohorts. Where univariate analyses indicated significant differences, pairwise comparison showed a significant increase in eye muscle area in the liquid MYLO and X2 MYLO treatment groups compared to controls (*P* = 0.009 and *P* = 0.037 respectively, Table 3, Table S3).

**Table 4:** Mean carcass traits of feedlot steers by treatment for control, MYLO liquid or dried supplement treatment groups. Data is shown as mean ± SEM.

	Control^1^	X1 MYLO^1^	X2 MYLO^1^	Liquid MYLO^1^	TRT *P*-value
**HSCW^2^, kg**	347.72 ± 2.74	350.89 ± 2.81	355.44 ± 2.70	348.54 ± 2.72	>0.05
**Lean Meat Yield, %**	52.26 ± 2.24	54.10 ± 1.98^a^	53.88 ± 2.16	54.71 ± 2.15	>0.05
**Ossification score**	129.81 ± 2.57	133.81 ± 2.57	130.20 ± 2.46	133.14 ± 2.48	>0.05
**pH**	5.47 ± 0.07	5.48 ± 0.05	5.47 ± 0.05	5.47 ± 0.07	>0.05
**Eye muscle area, cm**2	80.28 ± 0.85^a,b,c^	82.24 ± 0.87^a^	82.71 ± 0.84^a,b^	83.59 ± 0.84^a,c^	>0.05^a^
					**0.037** ^a,b^
					**0.008** ^a,c^
**Rib fat depth, mm**	10.21 ± 0.43	10.57 ± 0.44	11.11 ± 0.42	10.18 ± 0.42	>0.05
**MSA^3^ marble score**	408.87 ± 77.98	437.69 ± 92.72	421.54 ± 84.47	427.80 ± 83.67	>0.05
**MSA^4^ Index**	63.35 ± 0.21	63.53 ± 0.21	63.90 ± 0.20	63.46 ± 0.21	>0.05
**Total carcass value, AUD$**	$2259.36 ± 17.81	$2279 ± 18.27	$2300.49 ± 17.55	$2262.60 ± 17.68	>0.05

1 Control *n* = 62; X1 MYLO *n* = 65; x2 MYLO *n* = 63; Liquid MYLO *n* = 65.

2 Hot Standard Carcass Weight.

3 Meat Standards Australia.

4 Meat Standards Australia index is a number between 30 and 80 that predicts the eating quality of a beef carcase. The index is a weighted score based on eating quality of 39 primals related to other carcase measures (eg rib fat depth, marble score, ossification score, hump height, sex, HSCW, sex and HGP status) and is calculated for Achilles-hung carcasses with 5 days ageing. A carcase with a higher score will have higher eating quality.

a,b,c Within row means with different superscripts differ (*P* < 0.05).

Significant *P* values are shown in bold.

### Antemortem disease rates

Post-mortem offal examination found liver fluke (live or past infestation, *n* = 69), liver abscess (grade 1 or 2 where grade 1 is evidence of a single abscess and grade 2 is evidence of 2 or more abscesses, *n* = 47) liver adhesions (6), cirrhosis (1), telangectasia (10), lung abscess (9), pneumonia (2), enteritis (2), and skirt abscess (2). Liver abscess was observed with lung abscess in 8/9 steers presenting for that condition. The most prevalent health conditions (liver fluke–live and past; liver abscess, lung abscess and telangiectasia) are shown in Table 5. Statistical analysis failed to identify an influence of DFM treatment on the incidence of any of the reported conditions due to pen exerting a significant effect in this analysis.

**Table 5:** Mean prevalence of ante-mortem carcass health conditions of feedlot steers by treatment group.

Disease, % (*n*)	Control^1^	X1 MYLO^1^	X2 MYLO^1^	Liquid MYLO^1^	TRT, *P*-value
**Liver fluke**	24.2 (15)	20.0 (13)	30.2 (19)	33.8 (22)	>0.05
**Liver abscess**	19.4 (12)	18.5 (12)	22.2 (14)	12.3 (8)	>0.05
**Lung abscess**	3.2 (2)	1.5 (1)	7.9 (5)	1.5 (1)	>0.05
**Telangectasia**	3.2 (2)	3.1 (2)	4.8 (3)	4.6 (3)	>0.05

1 Control *n* = 62; X1 MYLO *n* = 65; x2 MYLO *n* = 63; Liquid MYLO *n* = 65.

## Discussion

There is increasing pressure for beef producers to achieve high yield with minimal environmental impact. One way to achieve this is to increase beef cattle production efficiency through targeted supplementation to increase growth rates through improved feed conversion. This is desirable for markets, such as the European Union, that do not accept imported carcasses finished using hormonal growth promotants. In addition, there is an increasing scrutiny on the use of in-feed ionophores, and the broader use of antimicrobials in livestock production, for which alternatives are now being sought. One option for both growth promotion largely through rumen pH stabilisation and as an alternative to in-feed ionophores, are live bacterial cultures known as probiotics or Direct-Fed Microbials (DFMs), that are increasingly being used in human and veterinary feed formulations.

The aim of this trial was to establish equivalence to, and efficacy of, a novel dried formulation of the DFM ‘MYLO’, compared with the commercially available liquid supplement. All MYLO supplemented cattle were compared to a control cohort that did not receive any DFM supplementation. Both Liquid MYLO and the X2 dried formulation showed increases in eye muscles area, and increased carcase values were also noted, a result of tangible economic interest.

LW gain or increased carcass performance are not commonly reported for DFMs, and rarely are the same treatments at different doses considered comparatively. Specifically, Opheim and colleagues (2023) report no effect of DFM treatment containing *Lactobacillus salivarius* in Angus cross-bred steers fed a steam-flaked corn finisher diet, potentially caused by a reduction in DMI in the treatment group, although an increase in rumen papillae number was observed. Another study by Gubbels and colleagues (2023) in long-fed cattle found no impact of DFM treatment on growth characteristics, carcass performance or health. Further, most studies and reviews of DFMs have noted a lack of effect on growth rates and carcass performance (Kenney et al. 2015; Mayer et al. 2022; Cusack 2024), making the findings in the current study worthy of further investigation. It is also important to note that this trial did not supplement growth performance with use of HGPs. This was to ensure that any growth characteristics were attributable to the DFM alone, without extraneous biological influences on growth performance.

The mechanism of action of the Lactobacillaceae in the rumen of supplemented cattle is unknown but prior studies with similar bacterial strains have identified antimicrobial and antifungal properties of *Lacticaseibacillus* species. *Lacticaseibacillus paracasei* has been shown to excrete low molecular weight substances with antimicrobial activity, particularly against known enteric pathogens (Reuben et al. 2019, 2020). *Lentilactobacillus buchneri* has been recently shown to inhibit the growth of yeasts in silage preparations (Nair et al. 2022) and to increase the availability of volatile fatty acids in treated silages, (Nair et al. 2020) potentially increasing fermentation stability and biodigestibility of the resultant feed. The combination of three DFMs in the MYLO™ formulation, specifically *Lacticaseibacillus paracasei*, *Lentilactobacillus buchneri* and *Lacticaseibacillus casei*, may therefore be conferring some antibacterial or bacteriostatic property to the rumen microbiome, potentially inhibiting the proliferation of pathogenic or other function-limiting bacterial species. Microbial strain differences may confer different growth properties when fed in controlled trial and commercial settings and a recent study by Bowman-Schnug and colleagues has reported improved health performance in feedlot steers fed a *Saccharomyces cerevisiae* fermentation product, suggesting that metabolites produced by DFMs may confer beneficial properties (Bowman-Schnug 2025). Therefore, it is possible that the specific combination of DFMs present in the MYLO supplement may confer preferential properties although larger trials would be required to confirm an effect.

A limitation of the current study was that sampling of rumen fluid was not undertaken, a process that could yield information on changes in the rumen microbiome in response to ingestion of the live DFM, persistence of the strains present in the MYLO supplements over time in the rumen, and also to provide insight into any secondary metabolites generated by the DFM species that could be conferring additional properties either to the diet or to the rumen microenvironment. Fluctuations in rumen microflora in feedlot cattle have been noted under standard introduction to grain feeding (Maslen et al. 2022) as well as the rumen microbiome profile influencing carcase weight in high versus low carcase weight Japanese steers (Sato et al. 2024). The potential for positive effects of the DFM on rumen microflora relative to both transition diets and whole of time on feed should be addressed in further studies with both the liquid and dried MYLO DFM formulation, particularly given the proposed longitudinal nature of exposure to DFM supplementation in feedlot steers.

No increase in disease incidence was noted in DFM-fed steers in this trial compared to their control counterparts, nor was there an increased prevalence of antemortem disease conditions noted. Incidence of liver abscesses are relatively common in grain-fed cattle, and exposure to MYLO DFM did not confer protective properties, or alternatively negatively impact health outcomes in any of the treatment groups. Liver abscesses in feedlot cattle are commonly caused by *Fusobacterium necrophorum subsp. necrophorum* (Herrick et al. 2022; Abbasi et al. 2024), a bacterium that is part of the normal rumen flora and thought to translocate to the liver via breaches in the rumen lining (Nagaraja and Chengappa 1998; Abbasi et al. 2024). As incidence of rumen acidosis in our trial was very low, it is not possible to confirm if the DFM exerted protected properties and larger cohort studies would be required to confirm a beneficial effect.

Overall, whilst the remit of this trial was to establish equivalence between two novel dried formulations of the MYLO DFM supplement and the current commercially available liquid formulation. We identified a modest positive beneficial effect on carcase performance, specifically eye muscle area, for a novel dried DFM at the higher concentration, MYLO when fed at a high concentration (2 × 10^10^ CFU/h/day), although a larger study would be required to unequivocally confirm this effect in a commercial setting. The mechanism of interaction of the MYLO supplements and feed type on rumen function and nutrient processing was not examined in this study, nor was persistence of the microorganisms in the animal. These could also be confirmed with additional experiments looking at the impact of DFM supplementation on rumen microstructure and nutrient bioavailability.

Further studies are required to determine the mechanism of action of high-concentration MYLO DFMs on rumen microbiome and persistence of DFM species post dosing, nutrient digestibility and feedlot performance at commercial scale. Evaluation of potential interactions of the DFM with diets varying in starch and fibre concentrations and effects on the health and production of feedlot cattle are also warranted for applicability under multiple operating conditions.

## Supplementary data

Supplementary data is available at *Translational Animal Science* online.

## Supplementary Material

txag033_Supplementary_Data

## Abbreviations

ADG: average daily gain
AUD: Australian dollar
CFU: colony forming unit
CP: crude protein
d: day
DOF: days on feed
DFM: direct-fed microbial
DM: dry matter
G:F: gain to feed ratio
EID: electronic identification
FCR: feed conversion ratio
g: gram
HSCW: hot standard carcass weight
kg: kilogram
L: litre
ME: metabolizable energy
MSA: Meat Standards Australia
NDF: neutral detergent fibre
NFAS: National Feedlot Accreditation Scheme (Australia)
Sem: standard error of the mean
TMR: traditional mixed ration
VID: visual identification
