# Supplementary material for: Comparison of dried and liquid direct-fed microbial (MYLO) on liveweight gain and carcass performance in feedlot cattle
Source: Transl Anim Sci. 2026 Mar 22;10:txag033. doi: 10.1093/tas/txag033 (PMC13044504; doi:10.1093/tas/txag033)
Supplement: txag033_Supplementary_Data [file txag033_supplementary_data.zip › Quinn_Cusack_Supplementary Table 1.docx]

**Supplementary Table 1**. Bonferroni Pairwise comparison of estimated marginal means of mean exit liveweight at 106 days on feed for steers fed a control diet without supplementation (control) or one of three MYLO DFM supplements (X1, X2 or Liquid). Pen was considered a random effect, and origin were a covariate, neither exerted an influence on the model. Significant differences (*p =* <0.05) are shown in **bold**.

| Treatment (I) | Treatment (J) | Mean Difference (I-J) | s.e.m. | Sig | 95% Confidence | |
| --- | --- | --- | --- | --- | --- | --- |
|  |  |  |  |  | Lower Bound | Upper Bound |
| Control | X1 | 2.901 | 6.897 | 0.828 | -15.445 | 21.247 |
|  | X2 | -9.006 | 6.992 | 0.778 | -27.604 | 9.593 |
|  | Liquid | 4.930 | 6.946 | 0.236 | -13.548 | 23.407 |
| X1 | Control | -2.901 | 6.897 | 0.828 | -21.247 | 15.445 |
|  | X2 | -11.907 | 6.856 | 0.062 | -30.144 | 6.330 |
|  | Liquid | 2.028 | 6.795 | 0.529 | -16.407 | 20.104 |
| X2 | Control | 9.006 | 6.992 | 0.778 | -9.593 | 27.604 |
|  | X1 | 11.907 | 6.856 | 0.062 | -6.330 | 30.144 |
|  | Liquid | 13.935 | 6.907 | **0.042** | -4.437 | 32.308 |
| Liquid | Control | -4.930 | 6.946 | 0.236 | -23.407 | 13.548 |
|  | X1 | -11.907 | 6.795 | 0.529 | -20.104 | 16.047 |
|  | X2 | -13.935 | 6.907 | **0.042** | -32.308 | 4.437 |
