# Supplementary material for: Comparison of dried and liquid direct-fed microbial (MYLO) on liveweight gain and carcass performance in feedlot cattle
Source: Transl Anim Sci. 2026 Mar 22;10:txag033. doi: 10.1093/tas/txag033 (PMC13044504; doi:10.1093/tas/txag033)
Supplement: txag033_Supplementary_Data [file txag033_supplementary_data.zip › Quinn_Cusack_Supplementary Table 2.docx]

**Supplementary Table 2**. Bonferroni Pairwise comparison of estimated marginal means of Average Daily Gain for steers fed a control diet without supplementation (control) or one of three MYLO DFM supplements (X1, X2 or Liquid). Pen was considered a random effect, and origin were a covariate, neither exerted an influence on the model. Significant differences (*p =* <0.05) are shown in **bold**.

| Diet (I) | Diet (J) | Mean Difference (I-J) | Std. Error | Sig | 95% Confidence Interval for Difference^a^ | |
| --- | --- | --- | --- | --- | --- | --- |
|  |  |  |  |  | Lower Bound | Upper Bound |
| C | X1 | -.104 | .062 | .574 | -.268 | .061 |
|  | X2 | -.151 | .062 | .097 | -.317 | .015 |
|  | L | -.018 | .062 | 1.000 | -.183 | .146 |
| X1 | X2 | -.048 | .062 | 1.000 | -.212 | .116 |
|  | C | .104 | .062 | .574 | -.061 | .268 |
|  | L | .085 | .061 | .987 | -.077 | .248 |
| X2 | X1 | .048 | .062 | 1.000 | -.116 | .212 |
|  | C | .151 | .062 | .097 | -.015 | .317 |
|  | L | .133 | .062 | .193 | -.031 | .297 |
| L | X1 | -.085 | .061 | .987 | -.248 | .077 |
|  | X2 | -.133 | .062 | .193 | -.297 | .031 |
|  | C | .018 | .062 | 1.000 | -.146 | .183 |
