# Supplementary material for: Comparison of dried and liquid direct-fed microbial (MYLO) on liveweight gain and carcass performance in feedlot cattle
Source: Transl Anim Sci. 2026 Mar 22;10:txag033. doi: 10.1093/tas/txag033 (PMC13044504; doi:10.1093/tas/txag033)
Supplement: txag033_Supplementary_Data [file txag033_supplementary_data.zip › Quinn_Cusack_Supplementary Table 3.docx]

**Supplementary Table 3**. Bonferroni pairwise comparison of carcase characteristics for steers fed a traditional mixed ration for 106D with or without MYLO supplementation. Pen was considered a random effect, and pen location were as a covariate, neither exerted an influence on the model. Significant differences (*p =* <0.05) are shown in **bold**.

| Dependent Variable | Diet code (I) | Diet code (J) | Estimated marginal mean Difference (I-J) | s.e.m. | Sig. | 95% Confidence | | |
| --- | --- | --- | --- | --- | --- | --- | --- | --- |
|  |  |  |  |  |  | Lower Bound | Upper Bound |  |
| Ossification | Control | X1 | -3.919 | 3.759 | 0.298 | -11.322 | 3.484 |  |
|  |  | X2 | -.397 | 3.412 | 0.907 | -7.117 | 6.323 |  |
|  |  | Liquid | -3.202 | 3.584 | 0.373 | -10.261 | 3.858 |  |
|  | X1 | Control | 3.919 | 3.759 | 0.298 | -3.484 | 11.322 |  |
|  |  | X2 | 3.522 | 3.664 | 0.337 | -3.694 | 10.739 |  |
|  |  | Liquid | .718 | 3.611 | 0.843 | -6.394 | 7.829 |  |
|  | X2 | Control | .397 | 3.412 | 0.907 | -6.323 | 7.117 |  |
|  |  | X2 | -3.522 | 3.664 | 0.337 | -10.739 | 3.694 |  |
|  |  | Liquid | -2.805 | 3.604 | 0.437 | -9.903 | 4.294 |  |
|  | Liquid | Control | 3.202 | 3.584 | 0.373 | -3.858 | 10.261 |  |
|  |  | X1 | -.718 | 3.611 | 0.843 | -7.829 | 6.394 |  |
|  |  | X2 | 2.805 | 3.604 | 0.437 | -4.294 | 9.903 |  |
| pH | Control | X1 | -.007 | .011 | 0.485 | -.028 | 0.013 |  |
|  |  | X2 | .010 | .010 | 0.276 | -.008 | 0.029 |  |
|  |  | Liquid | .001 | .010 | 0.902 | -.019 | 0.021 |  |
|  | X1 | Control | .007 | .011 | 0.485 | -.013 | 0.028 |  |
|  |  | X2 | .018 | .010 | 0.084 | -.002 | 0.038 |  |
|  |  | Liquid | .009 | .010 | 0.395 | -.011 | 0.029 |  |
|  | X2 | Control | -.010 | .010 | 0.276 | -.029 | 0.008 |  |
|  |  | X2 | -.018 | .010 | 0.084 | -.038 | 0.002 |  |
|  |  | Liquid | -.009 | .010 | 0.364 | -.029 | 0.011 |  |
|  | Liquid | Control | -.001 | .010 | 0.902 | -.021 | 0.019 |  |
|  |  | X1 | -.009 | .010 | 0.395 | -.029 | 0.011 |  |
|  |  | X2 | .009 | .010 | 0.364 | -.011 | 0.029 |  |
| RibFat depth | Control | X1 | -.368 | .648 | 0.570 | -1.645 | 0.908 |  |
|  |  | X2 | -.904 | .588 | 0.126 | -2.063 | 0.255 |  |
|  |  | Liquid | .022 | .618 | 0.972 | -1.195 | 1.239 |  |
|  | X1 | Control | .368 | .648 | 0.570 | -.908 | 1.645 |  |
|  |  | X2 | -.536 | .632 | 0.397 | -1.780 | 0.708 |  |
|  |  | Liquid | .390 | .623 | 0.532 | -.836 | 1.616 |  |
|  | X2 | Control | .904 | .588 | 0.126 | -.255 | 2.063 |  |
|  |  | X2 | .536 | .632 | 0.397 | -.708 | 1.780 |  |
|  |  | Liquid | .926 | .621 | 0.137 | -.298 | 2.150 |  |
|  | Liquid | Control | -.022 | 0.618 | 0.972 | -1.239 | 1.195 |  |
|  |  | X1 | -.390 | 0.623 | 0.532 | -1.616 | 0.836 |  |
|  |  | X2 | -.926 | 0.621 | 0.137 | -2.150 | 0.298 |  |
| Eye Muscle Area | Control | X1 | -1.958 | 1.278 | 0.127 | -4.476 | 0.559 |  |
|  |  | X2 | -2.428 | 1.160 | **0.037** | -4.713 | -0.143 |  |
|  |  | Liquid | -3.242 | 1.219 | **0.008** | -5.643 | -0.842 |  |
|  | X1 | Control | 1.958 | 1.278 | 0.127 | -.559 | 4.476 |  |
|  |  | X2 | -.470 | 1.246 | 0.707 | -2.924 | 1.984 |  |
|  |  | Liquid | -1.284 | 1.228 | 0.297 | -3.702 | 1.134 |  |
|  | X2 | Control | 2.428 | 1.160 | **0.037** | .143 | 4.713 |  |
|  |  | X2 | .470 | 1.246 | 0.707 | -1.984 | 2.924 |  |
|  |  | Liquid | -.814 | 1.226 | 0.507 | -3.228 | 1.599 |  |
|  | Liquid | Control | 3.242 | 1.219 | **0.008** | .842 | 5.643 |  |
|  |  | X1 | 1.284 | 1.228 | 0.297 | -1.134 | 3.702 |  |
|  |  | X2 | .814 | 1.226 | 0.507 | -1.599 | 3.228 |  |
| HSCW | Control | X1 | -3.267 | 4.119 | 0.428 | -11.379 | 4.845 |  |
|  |  | X2 | -6.641 | 3.739 | 0.077 | -14.004 | 0.723 |  |
|  |  | Liquid | -.825 | 3.927 | 0.834 | -8.560 | 6.910 |  |
|  | X1 | Control | 3.267 | 4.119 | 0.428 | -4.845 | 11.379 |  |
|  |  | X2 | -3.374 | 4.015 | 0.401 | -11.281 | 4.533 |  |
|  |  | Liquid | 2.442 | 3.956 | 0.538 | -5.350 | 10.234 |  |
|  | X2 | Control | 6.641 | 3.739 | 0.077 | -.723 | 14.004 |  |
|  |  | X2 | 3.374 | 4.015 | 0.401 | -4.533 | 11.281 |  |
|  |  | Liquid | 5.816 | 3.949 | 0.142 | -1.963 | 13.594 |  |
|  | Liquid | Control | .825 | 3.927 | 0.834 | -6.910 | 8.560 |  |
|  |  | X1 | -2.442 | 3.956 | 0.538 | -10.234 | 5.350 |  |
|  |  | X2 | -5.816 | 3.949 | 0.142 | -13.594 | 1.963 |  |
| Total carcase value | Control | X1 | -20.618 | 26.715 | 0.441 | -73.235 | 31.999 |  |
|  |  | X2 | -41.124 | 24.250 | 0.091 | -88.885 | 6.637 |  |
|  |  | Liquid | -3.235 | 25.475 | 0.899 | -53.410 | 46.939 |  |
|  | X1 | Control | 20.618 | 26.715 | 0.441 | -31.999 | 73.235 |  |
|  |  | X2 | -20.506 | 26.041 | 0.432 | -71.795 | 30.783 |  |
|  |  | Liquid | 17.383 | 25.663 | 0.499 | -33.161 | 67.926 |  |
|  | X2 | Control | 41.124 | 24.250 | 0.091 | -6.637 | 88.885 |  |
|  |  | X2 | 20.506 | 26.041 | 0.432 | -30.783 | 71.795 |  |
|  |  | Liquid | 37.889 | 25.617 | 0.140 | -12.565 | 88.343 |  |
|  | Liquid | Control | 3.235 | 25.475 | 0.899 | -46.939 | 53.410 |  |
|  |  | X1 | -17.383 | 25.663 | 0.499 | -67.926 | 33.161 |  |
|  |  | X2 | -37.889 | 25.617 | 0.140 | -88.343 | 12.565 |  |
| MSA Index | Control | X1 | -.186 | 0.315 | 0.555 | -0.806 | 0.433 |  |
|  |  | X2 | -.554 | 0.285 | 0.054 | -1.116 | 0.009 |  |
|  |  | Liquid | -0.115 | 0.300 | 0.701 | -.706 | 0.476 |  |
|  | X1 | Control | 0.186 | 0.315 | 0.555 | -.433 | 0.806 |  |
|  |  | X2 | -0.368 | 0.307 | 0.232 | -.971 | 0.236 |  |
|  |  | Liquid | 0.071 | 0.302 | 0.814 | -.524 | 0.666 |  |
|  | X2 | Control | 0.554 | 0.285 | 0.054 | -.009 | 1.116 |  |
|  |  | X2 | 0.368 | 0.307 | 0.232 | -.236 | 0.971 |  |
|  |  | Liquid | 0.439 | 0.302 | 0.147 | -.155 | 1.033 |  |
|  | Liquid | Control | 0.115 | 0.300 | 0.701 | -.476 | 0.706 |  |
|  |  | X1 | -0.071 | 0.302 | 0.814 | -.666 | 0.524 |  |
|  |  | X2 | -0.439 | 0.302 | 0.147 | -1.033 | 0.155 |  |
